# Aldehyde Dehydrogenase 1 (ALDH1) Is a Potential Marker for Cancer Stem Cells in Embryonal Rhabdomyosarcoma

**DOI:** 10.1371/journal.pone.0125454

**Published:** 2015-04-27

**Authors:** Kengo Nakahata, Shuichiro Uehara, Shimpei Nishikawa, Miyoko Kawatsu, Masahiro Zenitani, Takaharu Oue, Hiroomi Okuyama

**Affiliations:** 1 Departments of Pediatric Surgery, Osaka University Graduate School of Medicine, Osaka, Japan; 2 Frontier Science for Cancer and Chemotherapy, Osaka University Graduate School of Medicine, Osaka, Japan; Johns Hopkins University, UNITED STATES

## Abstract

Cancer stem cells (CSCs) are defined as a small population of cancer cells with the properties of high self-renewal, differentiation, and tumor-initiating functions. Recent studies have demonstrated that aldehyde dehydrogenase 1 (ALDH1) is a marker for CSCs in adult cancers. Although CSCs have been identified in some different types of pediatric solid tumors, there have been no studies regarding the efficacy of ALDH1 as a marker for CSCs. Therefore, in order to elucidate whether ALDH1 can be used as a marker for CSCs of pediatric sarcoma, we examined the characteristics of a population of cells with a high ALDH1 activity (ALDH1^high^ cells) in rhabdomyosarcoma (RMS), the most common soft tissue sarcoma in children. We used the human embryonal RMS (eRMS) cell lines RD and KYM-1, and sorted the cells into two subpopulations of ALDH1^high^ cells and cells with a low ALDH1 activity (ALDH1^low^ cells). Consequently, we found that the ALDH1^high^ cells comprised 3.9% and 8.2% of the total cell population, respectively, and showed a higher capacity for self-renewal and tumor formation than the ALDH1^low^ cells. With regard to chemoresistance, the survival rate of the ALDH1^high^ cells was found to be higher than that of the ALDH1^low^ cells following treatment with chemotherapeutic agents for RMS. Furthermore, the ALDH1^high^ cells exhibited a higher degree of pluripotency and gene expression of Sox2, which is one of the stem cell markers. Taken together, the ALDH1^high^ cells possessed characteristics of CSCs, including colony formation, chemoresistance, differentiation and tumor initiation abilities. These results suggest that ALDH1 is a potentially useful marker of CSCs in eRMS.

## Introduction

Cancer stem-like cells (CSCs) are defined as a small population of cancer cells with the properties of high tumor-initiating, self-renewal and differentiation functions [1]. In addition, CSCs are resistant to standard therapies, such as chemotherapy and radiotherapy, and thus responsible for tumor relapse after treatment as well as invasion and metastasis [2, 3].

Rhabdomyosarcoma (RMS) is the most common soft tissue sarcoma in children. Despite significant improvements in survival over the past few decades, more than one-third of RMS patients continue to die of the disease [4]. Patients with metastatic or refractory tumors exhibit a particularly severe prognosis [5]. Augmenting conventional regimens has not significantly improved survival, and research for CSCs of RMS is very important for improving the prognosis, as these cells are supposed to induce relapse and metastasis. Although CD133 (prominin-1) has been reported to be a marker for CSCs [6], it also exists on normal stem cells, and it is necessary to identify other markers for RMS.

Recent studies have demonstrated that aldehyde dehydrogenase 1 (ALDH1) is a marker for CSCs in adult cancers [7, 8, 9]. Although CSCs have been identified in many different types of pediatric solid tumors [10, 11], there are currently no studies regarding the efficacy of ALDH1 as a marker for CSCs in the field of pediatric oncology.

In this study, we hypothesized that a subpopulation of cells with a high ALDH1 activity (ALDH1^high^ cells) would display characteristics of CSCs in RMS and subsequently examined the characteristics of ALDH1^high^ cells in embryonal RMS (eRMS). We analyzed embryonal RMS cell lines using an ALDEFLUOR assay and found that the ALDH1^high^ cells had characteristics of CSCs, including colony formation, chemoresistance and tumor initiation abilities, and assessed the mRNA expression of ALDH1 isoforms, oncogene and stemness gene.

## Materials and Methods

### Cell line and cell culture

The human embryonal rhabdomyosarcoma cell line, RD and KYM-1 were obtained from ATCC (Manassas, VA, USA) and JCRB (Ibaraki, Japan), respectively. The cells were maintained in RPMI-1640 medium (Life Technologies, Carlsbad, CA, USA) supplemented with 1% penicillin/streptomycin and 10% fetal bovine serum (FBS) and cultured in a humidified 5% CO_2_ incubator at 37°C.

### ALDEFLUOR assay

The aldehyde dehydrogenase (ALDH) activity was detected using an ALDEFLUOR assay kit (StemCell Technologies, Vancouver, BC, Canada) according to the manufacturer’s protocol. Briefly, the cells were stained with bodipy-aminoacetaldehyde (BAAA) and incubated for 40 minutes at 37°C. A specific inhibitor of ALDH1, diemethylamino-benzaldehyde (DEAB), was used to control for background fluorescence. The stained cells were analyzed using the FACS Aria II (BD Biosciences, San Jose, CA, USA) and sorted into the ALDH1^high^ cells, which were detected on the green fluorescence channel (515–545 nm), and a subpopulation of cells with a low ALDH1 activity (ALDH1^low^ cells). The data were analyzed using the FACS DIVA software program (BD Biosciences). In order to exclude nonviable cells, 7-AAD(BD Biosciences)was added at a final concentration of 0.25 μg/ml.

### Colony formation assay

The sorted cells were suspended in 10 mL of RPMI-1640 and 10% FBS, and 1×10^4^ cells were plated in culture dishes with 3 mL of methylcellulose-containing RPMI-1640 supplemented with 10% FBS, according to the protocol of Rahadiani et al. [8]. The cells were stained with crystal violet (0.05% w/v), to visualize the colonies, and the number of colonies was counted after two months.

### Cell viability assay

To assess cell viability, the sorted cells were plated at 5×10^3^ cells per 96-well plates (Corning, Corning, NY, USA) for one day and then incubated with various concentrations of vincristine, cyclophosphamide and etoposide (Wako, Osaka, Japan) for three days. Subsequently, the degree of cell viability was investigated using the Cell Counting Kit-8 (Dojindo, Kumamoto, Japan) according to the manufacturer’s protocol.

### Adipocyte differentiation assay

For the adipocyte differentiation assay, the sorted cells were plated at a density of 1×10^4^ cells per 6-well plate (Corning, Corning, NY, USA)with 0.1% DMSO for three days. After eight days in RPMI, containing 1 μg/ml of insulin, 0.5 mM 3-isobutyl-1-methylxanthine, 10 mM dexamethasone, 1% penicillin/streptomycin and 10% FCS, neutral lipid accumulation was detected in cells fixed in 10% formaldehyde fixed cells using Oil Red O (Wako, Osaka, Japan) according to the protocol of Hwang et al. [12].

### Neurogenesis assay and immunofluorescence

For the neurogenesis assay, the sorted cells were plated at a density of 1×10^4^ cells per 6-well plate and treated with 100 nM ATRA (all-trans retinoic acid, Wako, Osaka, Japan). After three weeks, the cells were stained with NCAM (1/200) (123C3, monoclonal, Cell Signaling Technology, Danvers, MA, USA) via immunofluorescence according to the protocol of Walter et al. [6], and the nuclei were counterstained with DAPI (Dojindo).

For immunofluorescence staining, the sorted cells were fixed in 4% PFA and blocked in medium containing 3% BSA and 0.1% TritonX-100(Nacalai Tesque, Kyoto, Japan). The sorted cells were stained overnight at 4°C, and Alexa Fluor 488 goat anti-mouse IgG (1/1000) (A11001, Life Technologies) antibodies were used as secondary antibodies. All staining findings were analyzed with the BZ-9000 device (KEYENCE, Osaka, Japan).

### Xenograft transplantation

The sorted cells were collected and resuspended at a concentration of 10^2^–10^4^ cells per 100 μl of RPMI-1640 and then mixed with 100 μl of Matrigel (BD Biosciences, San Jose, CA, USA). The cell-Matrigel mixture was subsequently injected into the subcutaneous space in 4-week-old non-obese diabetic/severe combined immune-deficiency (NOD/SCID) mice (NOD. CB17-Prdkc^scid^/J, Charles River Laboratory, Yokohama, Japan) under general anesthesia. Tumor growth was monitored every other day and checked if the tumors were formed for two months.

The experiments were reviewed and approved by the Animal Experimentation Committee of Osaka University (permit number: 23-080-004), and conducted in accordance with institutional guidelines. All efforts were made to minimize suffering.

### Immunohistochemistry

The xenograft tumors of the mice and the clinical specimens were embedded in paraffin, fixed and analyzed for hematoxylin, Myogenin (1/200) (5FD, polyclonal, Santa Cruz Biotechnology, Dallas, TX, USA), Desmin (1/200) (H-76, polyclonal, Santa Cruz Biotechnology) and ALDH1 (1/200) (44/ALDH, monoclonal, BD Biosciences) staining using immunohistochemistry (IHC). As a secondary antibody, horseradish peroxidase (HRP)-labeled rabbit anti-mouse antibodies (Dako, Glostrup, Denmark) were used. The results of staining were visualized with the EnVision+ Single Reagent-Kit (Dako).

### Quantitative real-time PCR analysis

Quantitative real-time PCR was performed using the AB7900HT device (Applied Biosystems, Foster City, CA, USA) to determine the relative expression of the ALDH1 genes (ALDH1A1, ALDH1A2, ALDH1A3, ALDH1B1, ALDH1L1 and ALDH1L2), ABC transporter genes (ABCG2/BCRP, ABCB1/MDR1 and ABCA2), oncogene (c-Myc) and stemness marker (Sox2).

Total RNA was extracted using NucleoSpin RNA(Macherey-Nagel, Düren, Germany). Reverse transcription was carried out using the PrimeScript RT Master Mix (Takara, Shiga, Japan) according to the manufacturer’s protocol.

Real-time PCR reactions were performed in triplicates using the Platinum SYBR Green Super Mix with ROX (Life Technologies) on AB7900HT. The housekeeping gene, glyceraldehyde 3-phosphate dehydrogenase (GAPDH), served as an internal control, as it is expressed stably in different tissues. Table 1 lists the primers used for real-time PCR. The expression levels were calculated based on the 2^-ΔΔCT^ method.

**Table 1:** List of primers used for quantitative real-time PCR.

Target gene	Forward primer sequence	Reverse primer sequence
ALDH1A1	TGTTAGCTGATGCCGACTTG	TTCTTAGCCCGCTCAACACT
ALDH1A2	TGATCCTGCAAACACTGCTC	CTGGAGCTGGGTGGTAAGAG
ALDH1A3	TCTCGACAAAGCCCTGAAGT	TATTCGGCCAAAGCGTATTC
ALDH1B1	CTGGAGCTGGGTGGTAAGAG	CTTTCTCCACGGTTCTCTCG
ALDH1L1	ATCTTTGCTGACTGTGACCT	GCACCTCTTCTACCACTCTC
ALDH1L2	GCCTGGTCTCGTTACCAAAA	GCCACTTTCACCTCTTCAGC
ABCB1	GAGGAAGACATGACCAGGTA	CTGTCGCATTATAGCATGAA
ABCG2	ACCTGAAGGCATTTACTGAA	TCTTTCCTTGCAGCTAAGAC
ABCA2	AGATGGACAAGATGATCGAG	GCTTGTACTTCAGGATGAGG
c-Myc	GGAACGAGCTAAACGGAGCT	GGCCTTTTCATTGTTTTCCAATT
Sox2	CGAGTGGAAACTTTGTCGGA	TGTGCAGCGCTCGCAG
GAPDH	CAACGACCACTTTGTCAAGC	GGTGGTCCAGGGGTCTTACT

### Statistical Analysis

The statistical analyses were performed using the GraphPad Prism6 software package (GraphPad Software, La Jolla, CA, USA). *P* values of <0.05 were considered to be statistically significant according to Fisher’s exact test for xenograft transplantation and the Student’s *t*-test for the other experiments.

## Results

### Detection and characteristics of ALDH1^high^ cells as CSCs in RD and KYM-1 cells

**Fig 1:**
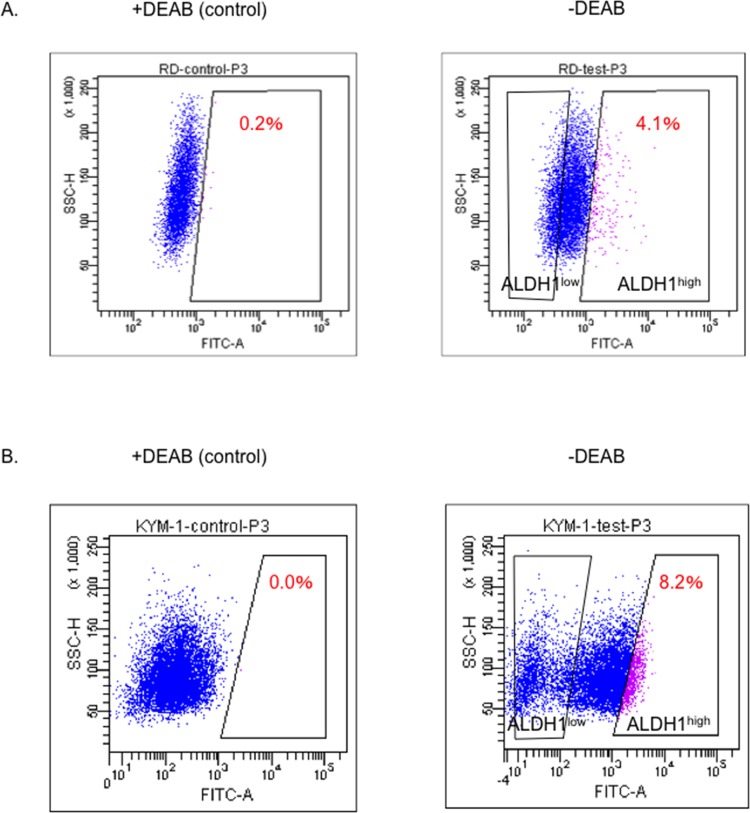
ALDH1^high^ cells were detected in the RD and KYM-1 cells. RD (A) and KYM-1 (B) cells were stained with DEAB (control: left panel) or without DEAB (right panel) after being stained with BAAA and then analyzed using FACS Aria II with the ALUDEFLUOR assay kit. The proportion of ALDH1^**high**^ cells was 3.9±0.26% in the RD cells and 8.2±0.14% in the KYM-1 cells. The mean ± SD was calculated from three independent experiments. All ALDH1^**high**^ cells and a subset of the ALDH1^**low**^ cells were sorted as shown in the right panels of Fig 1A and 1B.

We initially attempted to identify the ALDH^high^ cells in RD and KYM-1 cells using an ALDEFLUOR assay. In the RD cells, the ratio of the ALDH1^high^ cells stained with BAAA was 4.1%, while that of those stained with BAAA and DEAB as a negative control was 0.2%. Therefore, the genuine ratio of the ALDH1^high^ cells was 3.9% of all cultured RD cells (Fig 1A). Similarly, the ratio of the ALDH1^high^ cells was 8.2% in KYM-1 cells (Fig 1B).

**Fig 2:**
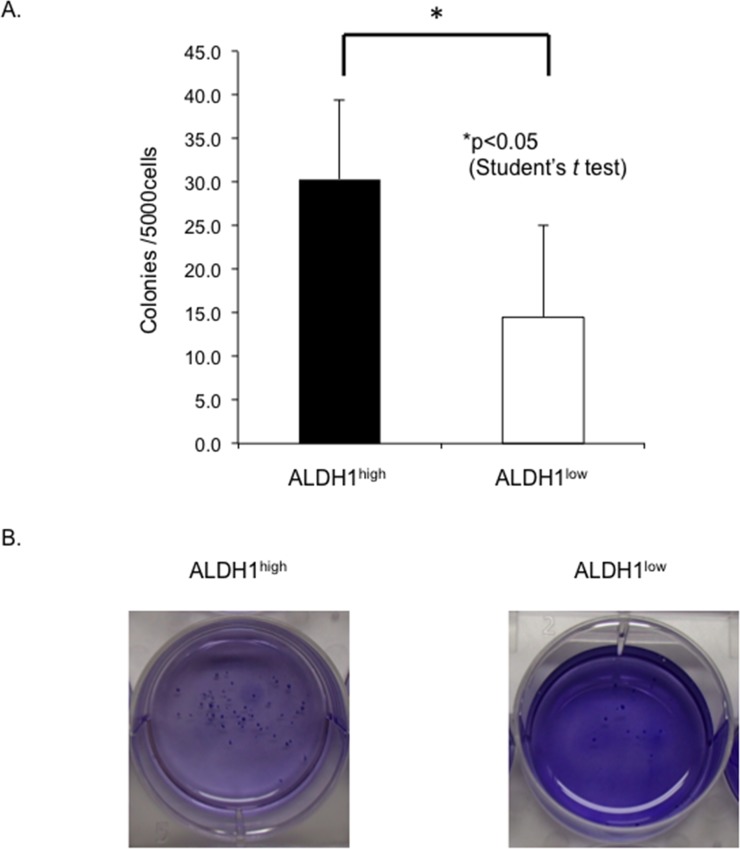
ALDH1^high^ cells have a higher capacity for self-renewal. A, Colony formation assay. The number of colonies that were macroscopically visible, derived from the ALDH1^**high**^ and ALDH1^**low**^ cells of RD cells, was counted and scored at two months after planting. The mean ± SD was calculated from three independent experiments (nine wells for ALDH1^**high**^ or ALDH1^**low**^ cells in total). The ALDH1^**high**^ cells formed significantly more colonies than the ALDH1^**low**^ cells. B, Representative images of colonies are shown in the left panel (ALDH1^**high**^) and right panel (ALDH1^**low**^).

Next, we performed a colony formation assay to document the self-renewal capacity of the ALDH1^high^ cells and ALDH1^low^ cells. Consequently, the number of colonies of ALDH1^high^ cells was higher than that of ALDH1^low^ cells (30.3 vs. 14.5 colonies/well, respectively, p<0.05) (Fig 2A and 2B). The ALDH1^high^ cells, therefore, had a greater colony formation ability than the ALDH1^low^ cells.

**Fig 3:**
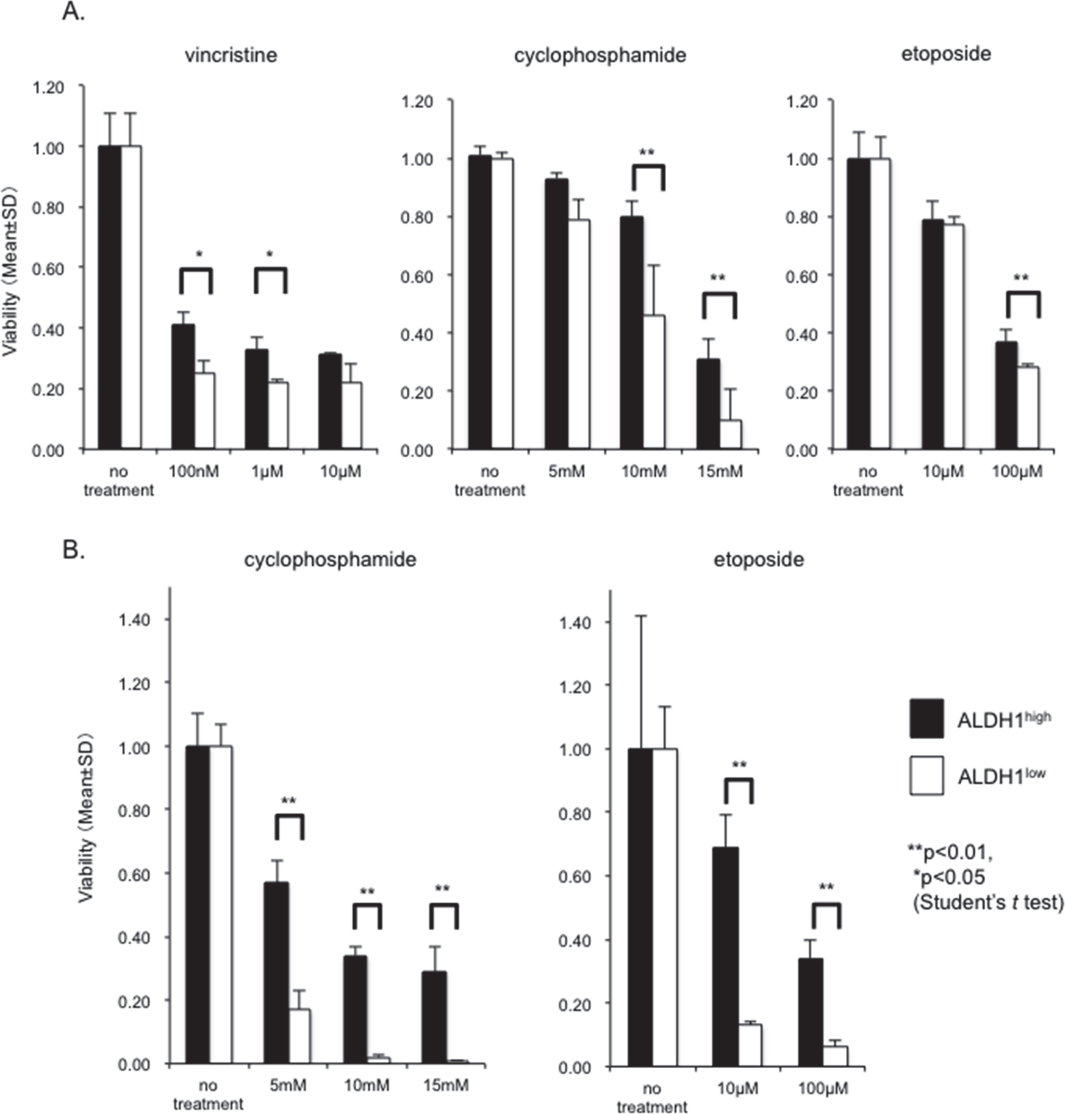
ALDH1^high^ cells show enhanced chemoresistance. The ALDH1^**high**^ and ALDH1^**low**^ cells of RD (A) and KYM-1 cells (B) were treated with 100 nM-10 μM vincristine, 5 mM-15 mM cyclophosphamide and 10 μM-100 μM etoposide and the cell viability was measured after 72 hours using a WST-8 assay. The viability of the “no treatment” cells was also measured as a control. The mean ± SD was calculated from triplicate wells of a representative experiment, and the data for three independent experiments are shown in the figure. The viability of the ALDH1^**high**^ cells was significantly higher than that of the ALDH1^**low**^ cells.

With regard to chemoresistance, we examined the survival rate of the ALDH1^high^ cells in the RD cells and KYM-1 cells cultured with vincristine, cyclophosphamide and etoposide using the Cell Counting Kit-8 (WST-8 assay). The viability of the ALDH1^high^ cells after culturing with vincristine, cyclophosphamide and etoposide was significantly higher than that of the ALDH1^low^ cells treated with these chemotherapeutic agents, suggesting that the ALDH1^high^ cells possessed a higher capacity for chemoresistance than the ALDH1^low^ cells (Fig 3).

**Fig 4:**
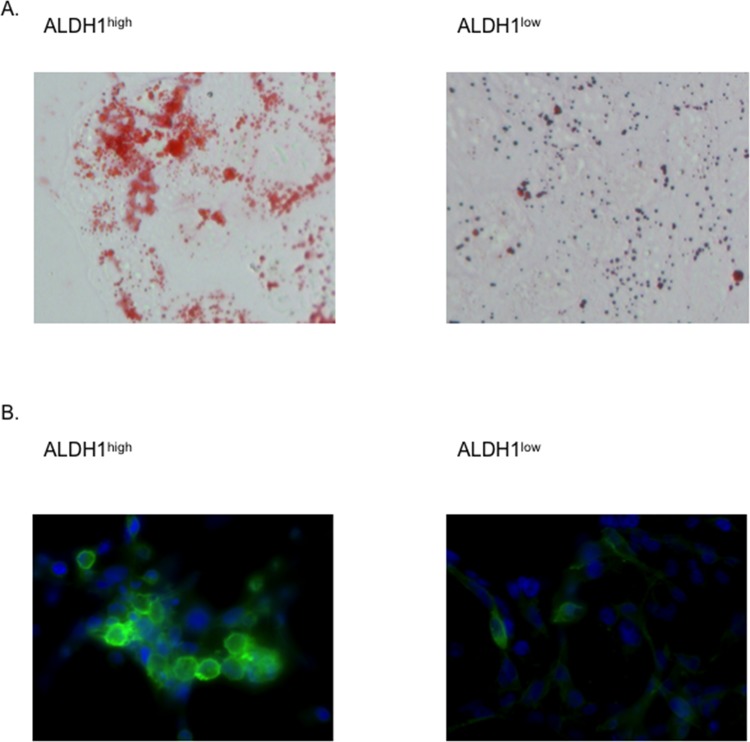
ALDH1^high^ cells possess a high capacity for pluripotency. A, Adipocyte differentiation assay. The ALDH1^**high**^ and ALDH1^**low**^ cells of RD cells were cultured in an adipocyte differentiation medium. The assay was repeated three times, and representative images of Oil Red O staining are shown in the left panel (ALDH1^**high**^) and in the right panel (ALDH1^**low**^) (×40). Lipid droplets of adipocytes stained red with Oil Red O. B, Neuronal cell differentiation assay. The ALDH1^**high**^ and ALDH1^**low**^ cells of RD cells were treated with 100 nM retinoic acid and stained for NCAM (green). The nuclei were counterstained with DAPI (blue). The assay was repeated three times, and representative images of immunofluorescence staining are shown in the left panel (ALDH1^**high**^) and in the right panel (ALDH1^**low**^).

As CSCs have been documented to exhibit pluripotency, we analyzed the differentiation ability of the ALDH1^high^ cells to adipocytes and neuronal cells in the RD cells. The ALDH1^high^ cells were found to be richer with lipid droplets stained with Oil Red O than the ALDH1^low^ cells (Fig 4A), and positive stainings were observed for NCAM in the ALDH1^high^ cells, indicative of neuronal differentiation (Fig 4B). These results indicate that the ALDH1^high^ cells of RD cells have the ability to differentiate into adipocytes and neuronal cells, indicating potential pluripotency.

**Fig 5:**
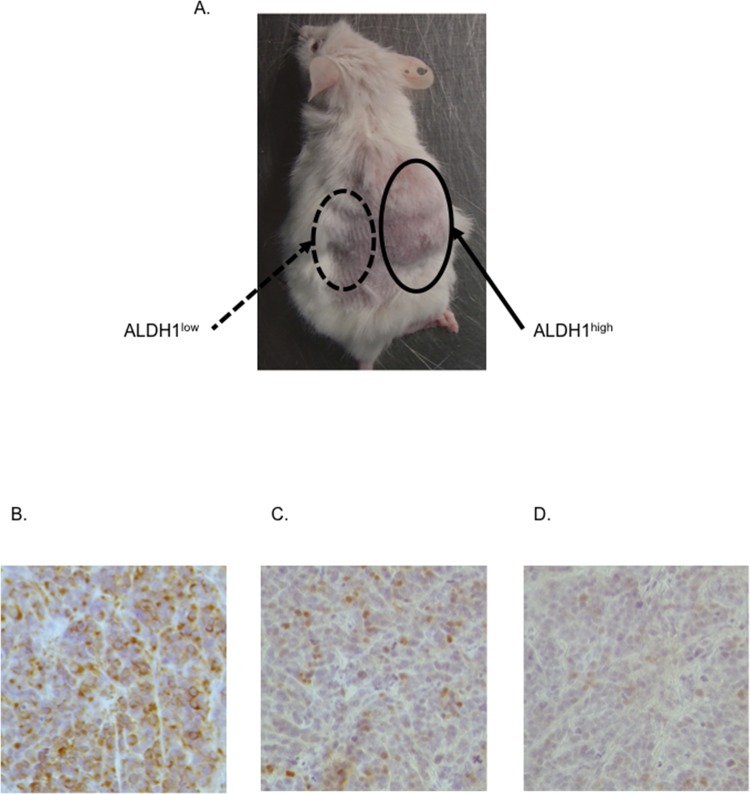
ALDH1^high^ cells have a high tumor-initiating ability. A, Representative image of a tumor-bearing NOD-SCID mouse. (right: ALDH1^**high**^ cells, left: ALDH1^**low**^ cells). B-D, Immunohistochemical (IHC) staining of the xenograft tumor sections. The sections were stained for hematoxylin, myogenic markers (Desmin (B), Myogenin (C)) and ALDH1 (D). The tumor cells were positive for Desmin and Myogenin while only a few cells were positive for ALDH1, suggesting that the ALDH1^**high**^ cells promoted rhabdomyosarcoma and were reconstituted to incorporate the full range of heterogeneity.

The tumor-initiating ability of the ALDH1^high^ cells in the RD cells was examined by injecting 1×10^2^, 1×10^3^ and 1×10^4^ ALDH1^high^ cells into NOD/SCID mice; ALDH1^low^ cells were also injected in the same manner. Consequently, tumor formation was observed in two of the seven mice injected with 1×10^3^ ALDH1^high^ cells and five of the six mice injected with 1×10^4^ ALDH1^high^ cells, while no tumors were found in the mice injected with ALDH1^low^ cells at either cell density (p<0.05, Table 2 and Fig 5A). These results show that the ALDH1^high^ cells have a significantly higher tumor-initiating ability than the ALDH1^high^ cells.

**Table 2:** The number of mice that formed RD tumors two months after inoculation.

	1×10^3^	1×10^4^	total
ALDH1^high^	2/7	5/6*	7/13**
ALDH1^low^	0/7	0/6	0/13

*p<0.05

**p<0.01 (Fisher’s exact test)

The ALDH1^high^ cells demonstrated a higher tumor-initiating ability than the ALDH1^high^ cells. The data from two independent experiments are summarized.

Next, we performed immunohistochemical (IHC) staining of the xenograft tumor sections of the ALDH1^high^ cells. These cells were stained positive for myogenic markers (Desmin, Myogenin) and ALDH1 (Fig 5B–5D). The tumor cells were positive for Desmin and Myogenin, while only a few cells were positive for ALDH1, suggesting that the ALDH1^high^ cells promoted rhabdomyosarcoma and were reconstituted to incorporate the full heterogeneity.

Taken together, we concluded that ALDH1^high^ cells are enriched with CSCs of RD and KYM-1 cells.

### The mRNA levels of ALDH1A3, ALDH1B1, ALDH1L2, ABCB1, ABCG2 and Sox2 were upregulated in the ALDH1^high^ cells of RD cells

**Fig 6:**
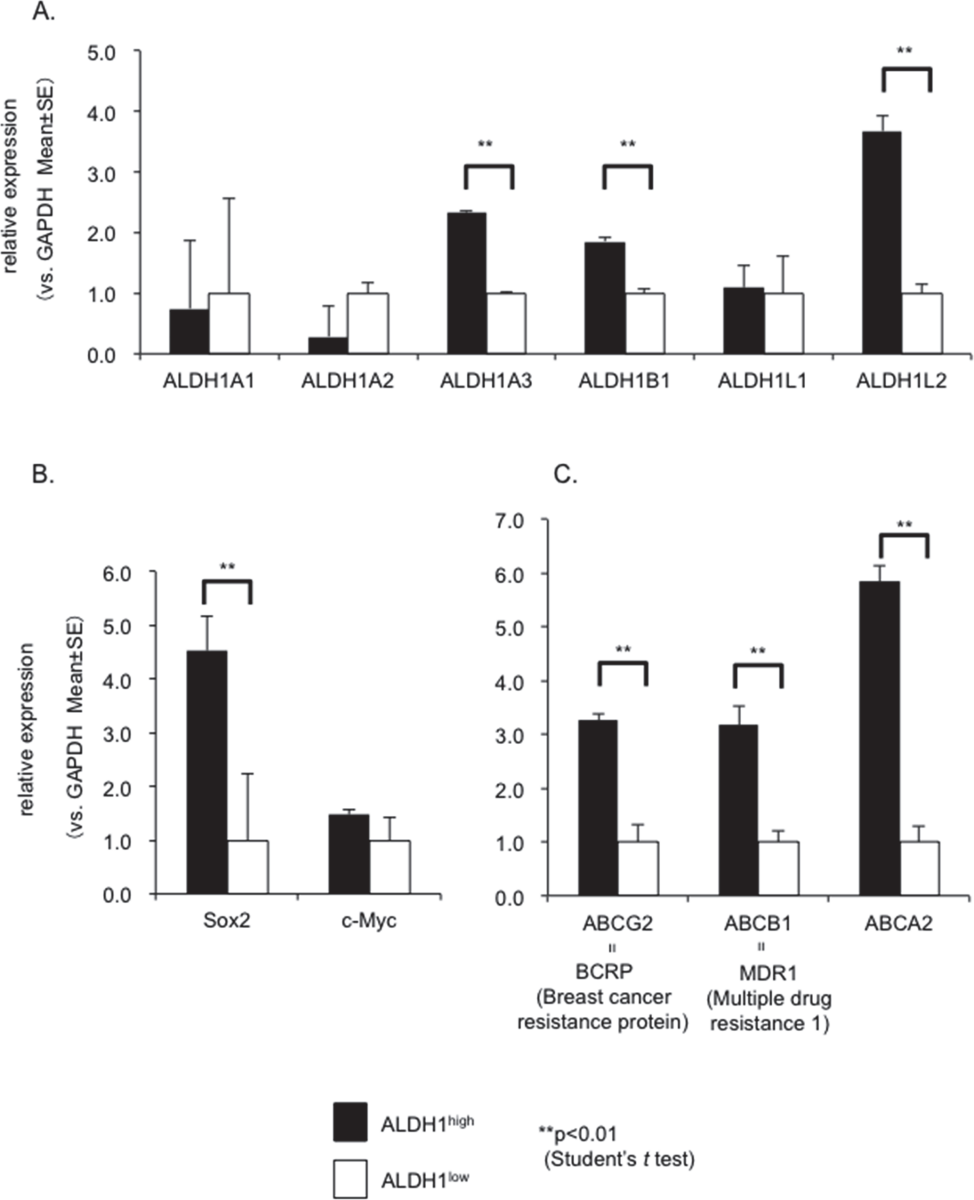
Upregulated mRNA in the ALDH1^high^ cells. A quantitative real-time PCR analysis was performed to evaluate the expression levels of ALDH1 (A), stemness markers (B) and ABC transporters (C). The mean ± SE was calculated from triplicate wells of a representative experiment, and the data for one of three independent experiments are shown in the figure. The expression of ALDH1A3, ALDH1B1, ALDH1L2, Sox2 and ABC transporters was significantly higher in the ALDH1^**high**^ cells than in the ALDH1^**low**^ cells (p<0.01).

Furthermore, we investigated the expression levels of several members of the ALDH1 family (ALDH1A1, ALDH1A2, ALDH1A3, ALDH1B1, ALDH1L1 and ALDH1L2) in the RD cells. As a result, the expression levels of ALDH1A3, ALDH1B1 and ALDH1L2, but not ALDH1A1, in the ALDH1^high^ cells were increased compared to that observed in the ALDH1^low^ cells (Fig 6A).

Next, we investigated whether ALDH1^high^ cells are enriched for the expression of genes thought to play important roles in stemness, such as c-Myc and Sox2. Quantitative real-time PCR showed an increased expression of Sox2 in the ALDH1^high^ cells relative to the ALDH1^low^ cells, whereas no significant increases were observed in the expression of c-Myc (Fig 6B).

The relative expression levels of three major drug transporters ABCG2/BCRP, ABCB1/MDR1 and ABCA2 were also examined. Interestingly, the ALDH1^high^ cells showed an increased expression of all transporters, suggesting that ALDH1^high^ cells have a higher capaciity for chemoresistance than ALDH1^low^ cells (Fig 6C).

## Discussion

The concept of CSCs has been long proposed [13]. CSCs are defined by two key characteristics, enhanced tumorigenicity and the capacity for self-renewal/differentiation [14]. Therefore, the isolated CSC population not only gives rise to de novo tumors with high efficiency, but also recapitulates the tumor with both CSC and non-CSC populations. In addition, most CSCs exhibit resistance to conventional anti-cancer therapies using chemotherapeutic agents and ionizing radiation [2]. To date, CSCs have been identified in various malignancies, including acute myeloid leukemia [15], brain tumors [16], hepatocellular carcinoma [17], breast cancer [7], lung cancer [18], pancreatic cancer [19] and ovarian cancer [8].

ALDH enzymes constitute a family of enzymes comprised of 19 isoforms localized in the cytoplasm, mitochondria and nucleus. ALDHs are responsible for oxidizing aldehydes to carboxylic acids. While many aldehydes play a critical role in physiological processes, such as vision, neurotransmission and embryonic development, most aldehydes are cytotoxic and must be detoxified, as reviewed by Marchitti et al [20].

A widely accepted method for identifying CSCs is based on detecting the enzymatic activity of ALDH1, a detoxifying enzyme responsible for the oxidation of intracellular aldehydes. The high activity of ALDH1 in CSCs has been used to isolate CSCs in different malignancies [6, 7, 8, 16, 18, 21, 22]. Therefore, ALDH1^high^ cells show features typically found in CSCs, including self-renewal, differentiation and high tumor-initiating abilities.

We confirmed that the ALDH1^high^ cells of the RD and KYM-1 cells possess characteristics of CSCs and, in another experiment using RMS-YM cells, an eRMS cell line, we examined the ALDH activity. Unlike that observed in the RD and KYM-1 cells, no ALDH1^high^ cells were detected. Next, as CSCs have been documented to have the ability to form spheres, we examined the sphere-forming ability of these cells. Consequently, the RD and KYM-1 cells formed large spheres, whereas the RMS-YM cells did not form spheres in serum-free medium (data not shown). These results strengthen our hypothesis that ALDH1^high^ cells possess characteristics of CSCs.

According to Lohberger et al. [23] and Awad et al. [24], ALDH1^high^ cells are typically isolated from human sarcoma cell lines (fibrosarcoma, liposarcoma, synovial sarcoma, chondrosarcoma, and Ewing’s sarcoma). Their study demonstrated that ALDH1^high^ cells are characterized by a high rate of proliferation, colony formation and expression of ABC transporter genes and stemness markers (β-catenin, Sox2 and Nanog). In the current study, ALDH1^high^ cells exhibited colony formation, as well as an increased gene expression of ABC transporters (ABCB1, ABCG2 and ABCA2) and stemness markers (Sox2). Therefore, our data suggest that ALDH1 is also a potential marker of CSCs in eRMS.

The ALDEFLUOR assay was developed to detect the activity of the ALDH1 isoform by successfully isolating viable hematopoietic stem cells from human umbilical cord blood [25] and has been reported to be specific to the ALDH1 isoform found in high abundance in these cells, ALDH1A1 [26]. However, while other individual ALDH isoforms do display some preferred substrate specificity, they also exhibit cross-reactivity, making it likely that the ALDEFLUOR assay will detect the ALDH1 activity of several ALDH isoforms expressed in the cells [27]. In the present study, the expression of ALDH1A3, ALDH1B1 and ALDH1L2 in the ALDH1^high^ cells was increased, while that of ALDH1A1, which is thought to play an important functional role in stem cells, was not increased in the ALDH1^high^ cells. Marcato et al. [28] reported that an increased ALDH activity in breast cancer stem cells is due to the effects of the isoform ALDH1A3, while Chen et al. [29] reported that ALDH1B1 is more profoundly expressed in adenocarcinomas than ALDH1A1, although the function and the cellular localization of ALDH1L2 remain unknown. Since our data are consistent with these findings, CSCs of RD cells may also have similar characteristics to those of epithelial tumor cells.

**Fig 7:**
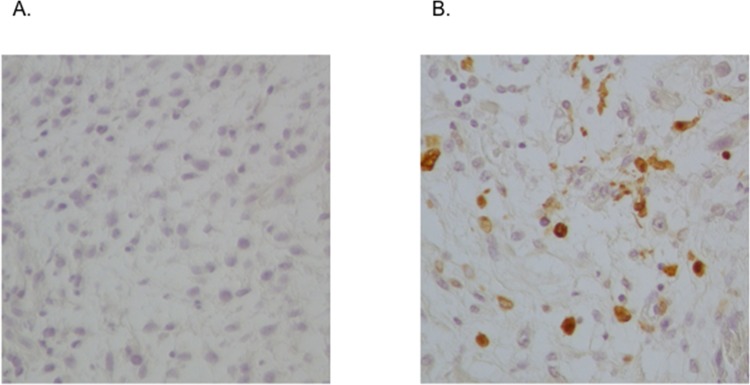
Comparison of the biopsy specimens obtained before chemotherapy and the resected specimens obtained after chemotherapy. The biopsy specimen of eRMS obtained prior to chemotherapy (A) and the resected specimens obtained after chemotherapy (B) stained positive for ALDH1 on immunohistochemistry. These specimens were taken from the vagina-originated eRMS tissue of a 1-year-old girl. The specimens resected after chemotherapy exhibited a greater cytoplasmic ALDH1 expression than the primary biopsy specimen.

A proposed mechanism for the chemoresistance of CSCs is based on the enhanced expression of ATP-binding cassette (ABC) transport proteins, which are responsible for drug efflux [30]. A high expression of ABC transporters in stem cells compared to non-stem cells results in relative resistance of the stem cells to the toxic effects of chemotherapy drugs. In this study, we analyzed the expression of three drug transporters (ABCG2/BCRP, ABCB1/MDR1 and ABCA2) of the ABC transporter family and found that all of the ABC transporters were upregulated in the ALDH1^high^ cells from RD. In addition, the ALDH1^high^ cells showed the increased resistance to vincristine, cyclophosphamide and etoposide, commonly used as the chemotherapeutic drugs of rhabdomyosarcoma. In fact, we performed the immunohistochemistry using the specimens of eRMS, resected before and after chemotherapy, and found that the specimens resected after chemotherapy exhibited a greater cytoplasmic ALDH1 expression than the primary specimen (Fig 7A and 7B).

These data suggest that the higher expression of the ABC transporter observed in the ALDH1^high^ cells causes the chemoresistance. In addition, these results are similar to side population cells (SP cells), which express various ABC transporters responsible for drug resistance, including ABCG2 (BRCP) [31]. Komuro et al. [32] reported SP cells were detected in RD using FACS with Hoechst dye. In fact, according to Yasuda et al. [33], SP cells and ALDH1^high^ cells overlap in ovarian cancer stem cells. Therefore, the populations of SP cells and the ALDH1^high^ cells may overlap in rhabdomyosarcoma as well.

This is the first study to document that ALDH1 may be used as a marker for RMS. We also used an alveolar RMS (aRMS) cell line (RH30) and examined the ALDH activity. Although ALDH1^high^ cells of RH30 cells were detected, unexpectedly the ALDH1^high^ cells of aRMS did not form colonies or spheres (data not shown). A likely explanation of this phenomenon is that the two major RMS subtypes arise from different cells of origin, given the substantial clinical and biological distinctions between them, as reported previously by Pressey et al. [34]. We are currently planning to examine whether ALDH1 can be used as a marker in other aRMS cell lines.

In recent years, research on induced pluripotent stem cells (iPSCs) has made rapid progress and has been applied to the field of oncology. Oshima et al. [35] reported the generation of induced CSCs (iCSCs) by introducing defined factors (Oct3/4, Sox2 and Klf4) into human colorectal cancer cell lines. As a result, the expression levels of ALDH1 were increased in the iCSCs. Therefore, ALDH1 may be related to CSC properties and function as a helpful tool for research on CSCs. Although the theory of cancer stem cells remains controversial [36], our results support the existence of CSCs in eRMS, and we believe that ALDH1 may be useful for detecting CSCs as a therapeutic target.

In conclusion, we confirmed that the ALDH1^high^ cells of eRMS possess characteristics of CSCs, including colony formation, chemoresistance and tumor initiation abilities. These results suggest that ALDH1 is a potentially useful marker of CSCs in eRMS.
